# Prevalence of Dietary Modification and Supplement Use in Patients with Metastatic Renal Cell Carcinoma Receiving Systemic Therapy

**DOI:** 10.3390/nu16111630

**Published:** 2024-05-26

**Authors:** Hedyeh Ebrahimi, Dena Battle, Nicholas J. Salgia, Zeynep B. Zengin, Nazli Dizman, Luis Meza, Cristiane D. Bergerot, Regina Barragan-Carrillo, JoAnn Hsu, Daniela Castro, Benjamin Mercier, Neal Chawla, Xiaochen Li, Abhishek Tripathi, Sandy T. Liu, Alex Chehrazi-Raffle, Ulka Vaishampayan, Michael D. Staehler, Sumanta K. Pal

**Affiliations:** 1 Department of Medical Oncology & Experimental Therapeutics, City of Hope Comprehensive Cancer Center, Duarte, CA 91010, USA; hebrahimi@coh.org (H.E.); zeynepbusrazengin@gmail.com (Z.B.Z.); luis.mezacontreras@yale.edu (L.M.); rbarragancarril@coh.org (R.B.-C.); johsu@coh.org (J.H.); dacastro@coh.org (D.C.); bmercier@coh.org (B.M.); nchawla@coh.org (N.C.); xiaoli@coh.org (X.L.); atripathi@coh.org (A.T.); achehraziraffle@coh.org (A.C.-R.); 2 Kidney Cancer Research Alliance (KCCure), Alexandria, VA 22314, USA; dena@kccure.org; 3 Department of Immunology, Roswell Park Comprehensive Cancer Center, Buffalo, NY 14203, USA; nicholas.salgia@roswellpark.org; 4 Jacobs School of Medicine and Biomedical Sciences, University at Buffalo, Buffalo, NY 14203, USA; 5 Department of Internal Medicine, MD Anderson Cancer Center, Houston, TX 77030, USA; ndizman@mdanderson.org; 6 Oncoclinicas&Co—Medica Scientia Innovation Research (MEDSIR), Sao Paulo 04543906, Brazil; crisbergerot@gmail.com; 7 City of Hope Orange County Lennar Foundation Cancer Center, Irvine, CA 92618, USA; sanliu@coh.org; 8 Department of Medicine/Oncology, Rogel Cancer Center, University of Michigan, Ann Arbor, MI 48109, USA; vaishamu@med.umich.edu; 9 Department of Urology, University Hospital Munich-Grosshadern, Ludwig-Maximilian University, 80539 Munich, Germany; michael.staehler@med.uni-muenchen.de

**Keywords:** renal cell carcinoma, dietary supplements, probiotics, diet therapy

## Abstract

Many patients diagnosed with cancer adopt dietary changes and supplement use, and a growing body of evidence suggests that such modifications can affect outcomes to cancer therapy. We sought to assess the prevalence of these practices and the surrounding physician-patient dialogue among patients with metastatic renal cell carcinoma. An online survey was administered by Kidney Cancer Research Alliance (KCCure), interrogating dietary modification patterns, supplement usage, out-of-pocket expenditure related to supplements, and patients’ views toward alternative medicine practices. Patients with metastatic renal cell carcinoma receiving combination therapy were actively solicited. In total, 289 unique responses were collected. The most common first-line treatments were nivolumab/ipilimumab (32.4%) and axitinib/pembrolizumab (13.1%). Within the cohort, 147 (50.9%) started using supplements following diagnosis of renal cell carcinoma; the most utilized supplements were probiotics, cannabidiol (CBD) oil/marijuana, and Vitamin C, reported by 70 (47.6%), 61 (41.4%), and 54 (36.7%), respectively. Dietary modifications following cancer diagnosis were reported by 101 (34.9%) respondents, of which 19.8% followed the Mediterranean diet and 18.8% adopted a ketogenic diet. Most respondents (71.3%) noted that they consistently report supplement usage to their physicians. A substantial proportion of patients with metastatic renal cell carcinoma utilize dietary modification and supplements as an adjunct to antineoplastic therapy. Considering the widespread adoption of these practices and the reported effects on cancer treatment, it is crucial for healthcare providers to engage in discussions with patients regarding supplement use.

## 1. Introduction

The prognosis of metastatic renal cell carcinoma (mRCC) has improved markedly over the course of the past several decades, owing largely to the advent of two classes of therapies: (1) vascular endothelial growth factor (VEGF)-directed agents, and (2) immune checkpoint inhibitors [1]. VEGF-directed therapies either abrogate signaling through binding of circulating VEGF (bevacizumab) or through inhibition of its cognate receptor (cabozantinib, lenvatinib, axitinib, and others) [2,3,4]. Commonly used checkpoint inhibitors in mRCC block signaling through either programmed death-1 (PD-1) or cytotoxic T-lymphocyte antigen 4 (CTLA4) [5]. Ahead of the introduction of these therapies, the median survival of mRCC was often quoted at approximately 1 year [6]. In contemporary studies using front-line combinations of VEGF-directed agents with checkpoint inhibitors or dual checkpoint inhibitor therapy, a median survival in excess of 4 years has been observed [7,8].

While improved prognosis and expanding treatment options for mRCC is a source of inspiration for many patients, the disease remains terminal for the majority. As a consequence, many patients turn to alternative strategies in the hopes of enhancing their outcomes. Such alternative strategies may encompass non-pharmacologic methods (e.g., exercise, meditation, and acupuncture) or pharmacologic approaches (e.g., herbs, vitamins, and minerals). We use the term “dietary supplements” herein to encompass the latter. The use of dietary supplements is pervasive not just among cancer patients but among healthy patients as well. The benefit in cancer outcomes is suspect. In the US-based National Health and Nutrition Examination Survey (NHANES) study, 30,958 adults were surveyed regarding dietary habits [9]. The majority of respondents (51%) reported use of dietary supplements, with 38% reporting use of multivitamins. With 6 years of follow-up, there was no reduction in cancer-related mortality: in fact, among patients taking calcium supplements, mortality was increased (hazard ratio [HR] 1.62, 95% CI, 1.07–2.45).

The literature around dietary supplement use among cancer patients is complex. A meta-analysis encompassing 32 large studies suggested that between 64 and 81% of cancer survivors use dietary supplements, with 14–32% of patients initiating these agents after a cancer diagnosis [10]. Rates of utilization were highest among patients with breast cancer, and multiple studies cite a higher rate of use among women as compared to men [11,12,13]. While questionnaire studies among patients consistently show high rates of dietary supplement use, up to 68% of treating physicians may be unaware of the practice, an alarming statistic [10]. Without systematic review and characterization of dietary supplements in the clinic, prior studies have shown that patients may experience harmful drug–supplement interactions or be taking agents that could compromise their long-term outcome [14].

Unlike in more prevalent cancer types (e.g., breast, lung, or prostate cancer), to our knowledge, there has been limited formal study of dietary intervention and supplement use among patients with RCC. To address this, we performed a survey study of a large online patient community of patients with RCC. Our results are focused specifically on those patients with metastatic disease on active systemic therapy.

## 2. Materials and Methods

The survey was developed by the Kidney Cancer Research Alliance (KCCure), a US-based non-profit patient advocacy forum, with multidisciplinary representation from a surgeon (MS), medical oncologist (UV), and patient advocate (DB). A committee with specific interest in microbiome research added questions pertaining to dietary habits and supplement usage (SKP, ND). A separate group of patient advocates initially evaluated the survey for ease of interpretability. The survey contained questions regarding patient demographics, including sex, age, race, country of residence, living area, and education level. Next, patients were asked about treatment-related information, consumption of supplements following cancer diagnosis, types of supplements utilized as a result of cancer diagnosis, dietary modifications following cancer diagnosis, views toward alternative medicine, frequency of sharing information about supplement use with their physician, and monthly out-of-pocket costs for supplements (see Appendix A for full survey).

The survey was distributed between July and September 2022 to a patient mailing list maintained by the KCCure and through online social media platforms (specifically, Facebook and Twitter). The system prohibited multiple responses from the same patient via anonymized IP address tracking. Responses from patients who (1) self-reported an mRCC diagnosis and (2) were receiving a checkpoint inhibitor-based systemic therapy regimen at the time of the survey were included for analysis.

### Statistical Analysis

Throughout this work, qualitative variables are provided as the number (percentage), and age is reported as the median (range: minimum–maximum). Descriptive statistics were used to report the frequency. The Student’s *t*-test and Chi-square test were used to compare sociodemographic characteristics between participants. Statistical analyses were conducted using R Statistical Software, version 4.3.0. (R Foundation for Statistical Computing, Vienna, Austria). All tests were two-sided, and an alpha level of 0.05 was considered for statistical significance.

## 3. Results

### 3.1. Characteristics of Patients

The survey was distributed to 1532 individuals, of which 1062 patients with RCC completed the survey. Among participants, 289 (27.2%) met the inclusion criteria of undergoing systemic therapy with a combination of dual checkpoint inhibitors or checkpoint inhibitors plus VEGF-directed agents for metastatic disease. Thus, our study population consisted of 143 (49.5%) females and 145 (50.2%) males, while 1 (0.3%) respondent preferred not to disclose their sex. The median age of the total inclusion cohort was 61 (range: 20–85 years). The majority (86.9%) of patients were residents of the United States, followed by Canada (3.1%). In this study, 269 (91.0%) respondents identified as white and 153 (52.9%) respondents had completed a bachelor’s degree or higher. The most commonly reported first-line treatments were nivolumab/ipilimumab (32.4%) and axitinib/pembrolizumab (13.1%). Detailed characteristics of respondents are presented in Table 1.

### 3.2. Supplement Use in Patients with Metastatic Renal Cell Carcinoma

Within the studied cohort, 147 (50.9%) respondents started using supplements following the diagnosis of mRCC. There were no statistically significant associations between the initiation of supplements following cancer diagnosis and age, sex, living area, or education. Among patients who began using supplements, 70 (47.6%) started using probiotics and 61 (41.4%) initiated cannabidiol (CBD) oil or marijuana subsequent to their cancer diagnosis. Table 2 presents the type and frequency of supplement use amongst respondents.

**Figure 1:**
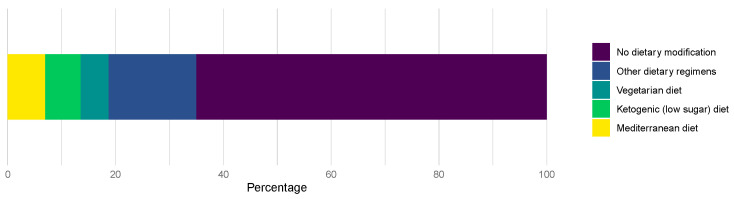
Dietary modification following the diagnosis of RCC.

Furthermore, 101 (34.9%) patients reported dietary modification after diagnosis of mRCC. The Mediterranean diet was the most common (19.8%) newly adopted regimen among patients, followed by the ketogenic diet (18.8%%) (Figure 1).

When patients were asked about the frequency of sharing information about supplement use with their physician, 206 patients (71.3%) indicated they always shared relevant information with their physician, 33 patients (11.4%) reported they usually/sometimes did so, and 8 patients (2.8%) reported rarely or never sharing information on supplement use with their provider. Notably, 42 patients (14.5%) preferred not to respond to this question. Survey prompts also assessed the out-of-pocket cost attributed to supplements and over-the-counter products not covered by insurance for patients with mRCC. Among the respondents, 103 patients (35.6%) spent less than $100 per month, 41 patients (14.2%) spent between $100 and $250 per month, and 24 patients (8.3%) spent more than $250 per month for supplements and over-the-counter products following mRCC diagnosis.

**Figure 2:**
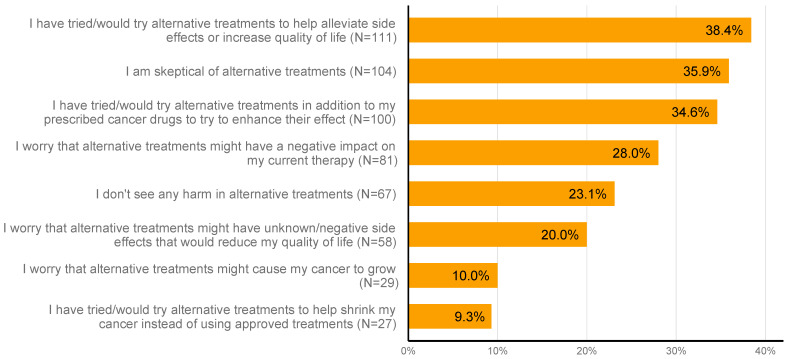
Respondents’ perspectives on supplements and alternative medicine.

Additionally, participants’ views toward alternative medicine were interrogated in this study. Notably, 111 patients (38.4%) indicated that they have tried or would try alternative medicine approaches to help alleviate the side effects of their systemic treatment or increase their quality of life. While 104 respondents (36.0%) were skeptical toward alternative medicine, 27 (9.3%) mentioned that they have tried or would try alternative medicine to assist their cancer shrinkage instead of using approved treatments. Detailed results of respondents’ views toward alternative treatment are presented in Figure 2.

## 4. Discussion

To our knowledge, the results herein represent the first study to systematically characterize the practice of supplement use within a population of patients diagnosed with mRCC. Our study identifies that most patients with mRCC use dietary supplements alongside formally prescribed anti-cancer therapy. Use of dietary supplements was prompted by the diagnosis of cancer in most cases, and the most frequently utilized dietary supplements included probiotics, cannabinoids, and vitamin C. Perspectives on dietary supplements varied among those surveyed, with roughly similar proportions of patients expressing optimism around improved clinical outcome and, on the contrary, concern around potential side effects. Reassuringly, most patients appear to maintain open communication with their treating physicians surrounding supplement use.

In the context of mRCC, the clinical impact of most dietary supplements is poorly understood. With the cornerstone of front-line therapy being immune checkpoint inhibitors, there is an emerging literature around how certain dietary interventions may impact patient outcomes in RCC and other tumor types with this class of therapy. Spencer and colleagues performed a detailed assessment of 128 patients with melanoma receiving checkpoint inhibitors: in this population, a high-fiber diet and no use of probiotics were associated with improved outcomes [15]. In preclinical models, the authors also demonstrated that low fiber intake or untailored probiotics were associated with impaired response to anti-PD-1 therapy and posit that such findings are driven by decreases in interferon-γ-expressing T cells in the tumor microenvironment. While untailored probiotics may hinder outcomes with checkpoint inhibitors, prospective trials combining live bacterial products (LBPs) with immune checkpoint inhibitors have been undertaken by our group and others, specifically with the intent of augmenting immunotherapy responses. The LBP CBM588 is a strain of *Clostridium butyricum*: once ingested, this LBP propagates in the lower gastrointestinal tract and prompts the release of butyrate, a short-chain fatty acid postulated to increase the recruitment of Th17 cells and other anti-tumor immune populations to the tumor microenvironment [16,17]. In two separate, phase I randomized trials, CBM588 has been shown to augment the activity of both cabozantinib/nivolumab and nivolumab/ipilimumab [18,19]. It is thus foreseeable that an evidence-based approach could be used to augment therapy via dietary supplementation, although further study is needed to validate this.

Furthermore, clinical anecdotes and preclinical studies have identified the potential for nutritional supplements to enhance immunotherapy outcomes in patients with cancer. Camu camu, for example, is a fruit native to Brazil and Peru, of which the active compound is castalagin. Recent work demonstrated that oral supplementation of castalagin enriched for bacterial species previously associated with immunotherapy response, improved the ratio of effector-to-regulatory T cells in the tumor microenvironment, and induced systemic metabolic changes, all of which associated with a biological program sufficient to bypass resistance to anti-PD1 immunotherapies in human xenograft models [20] Clinically, a recent report has also identified two patients with immunotherapy-refractive metastatic melanoma who went on to achieve durable responses to immunotherapy rechallenge after the addition of a camu camu prebiotic supplement [21]. Based on these compelling data, our group has initiated a randomized phase I trial to test camu camu in combination with nivolumab (anti-PD-1) plus ipilimumab (anti-CTLA-4) compared to nivolumab/ipilimumab alone for patients with treatment-naïve metastatic renal cell carcinoma [22].

While the literature around microbiome modulation is scant, there is even less literature to support the use of cannabinoids or vitamins in mRCC. In a study including 140 patients receiving nivolumab for mRCC, 51 patients (36%) were noted to have concurrent use of cannabis [23]. Interestingly, cannabis use was associated with an impaired response rate on multivariate analysis (38% versus 16%; *p* = 0.016), but no impact on progression-free survival (PFS) or overall survival (OS) was observed. Although the pathogenesis of this is unclear, several preclinical studies do point to both cannabinoid receptors 1 and 2 as being associated with RCC progression [24,25]. The recent announcement by the United States Drug Enforcement Administration to reclassify marijuana from a Schedule I to a Schedule III compound will allow for improved study of the interactions between cannabinoids and tumors, including RCC. Conflicting evidence exists around the role of vitamin C in RCC (the most commonly used vitamin supplement in our survey). Although meta-analyses point to an inverse relationship between vitamin C intake and the development of RCC, there is no data addressing the impact on metastatic disease [26]. Thus, future work on Vitamin C’s impact on RCC tumor growth and progression, alongside its interaction with anti-cancer therapies utilized in RCC, is imperative.

A modest proportion of patients in our series expressed concern over harm from alternative treatments. These concerns are warranted: for instance, a survey of 1081 cancer survivors from France identified that 18% of individuals taking dietary supplements had potentially harmful patterns of intake [14]. These practices included the consumption of phytoestrogens among patients with hormonally driven cancers (e.g., breast or prostate cancer) or the use of vitamin E with anticoagulants or antiplatelet agents (thereby enhancing bleeding risk). Studies evaluating the rates of drug interactions between dietary supplements and prescription medications in cancer patients have produced varying results, suggesting that interactions may occur in the range of 12 to 60% of patients [27,28]. These datasets highlight the importance of reviewing dietary supplement use with healthcare providers. Although some studies have pointed to low rates of information sharing between patients and providers, our study suggested that the majority of patients were open to discussing supplement use with their physicians [10].

Our study has multiple limitations. First, the survey was administered via online platforms and was open to any patients with a self-reported diagnosis of RCC. Thus, it is challenging to define an absolute denominator of patients to whom the survey was available. The population of respondents was representative of a typical RCC population based on many demographic features (i.e., age), but the gender distribution was equal (whereas RCC tends to have a 2:1 male predominance at the population level), and the declared race was largely white (91%), with relatively few minorities represented [29]. Patients were highly educated, with the majority of patients achieving a bachelor’s degree; the socioeconomic factors attached to this could have implications for the patterns of dietary supplement use observed (e.g., more affluent patients may have more resources to purchase these products). Reliance on self-reporting also makes it challenging to verify certain clinical characteristics, such as clinical stage or treatment status. Although our survey was quite detailed, we did not have the ability to capture certain detailed elements like supplement dose; therefore, although we presume that certain dietary supplements (e.g., vitamin C or E) might be used in excess, it is possible that patients are simply adhering to recommended daily values.

## 5. Conclusions

The data provided herein provide a first glimpse at dietary supplement use among patients with mRCC. Among nearly 300 patients with mRCC profiled herein, the majority of patients noted nutritional supplement use in addition to their checkpoint inhibitor-based systemic therapy. Furthermore, most patients maintained an open dialogue regarding supplement use with their providers. Given the prevalence of supplement use in the mRCC patient population and the potential risks posed by it, this dialogue between oncology providers and patients around dietary supplement use is critical to ensure patient safety and optimize positive therapeutic outcomes. Further work will seek to expand on these conversations to better understand both patient and provider perspectives on supplement use as an adjuvant to systemic anti-cancer therapies. The interest that patients express in dietary supplements is also justification for more formal study of their use, an avenue of research exploration that has only recently emerged. Together, our results represent the first interrogation of supplement use in a cohort of patients with mRCC receiving systemic therapy and open multiple avenues for future study to best optimize nutritional supplements and their interactions with anti-cancer therapy with the goal of improving patient outcomes in this aggressive disease setting.

## Figures and Tables

**Table 1:** Patient characteristics.

Demographic Characteristics (*n* = 289)		
Age: median (range)		61 (20–85)
Sex: number (%)	Female	143 (49.5%)
	Male	145 (50.1%)
	Prefer not to disclose	1 (0.3%)
Race: number (%)	White	263 (91.0%)
	Hispanic	11 (3.8%)
	Other	15 (5.2%)
Living area: number (%)	Urban	61 (21.1%)
	Suburban	133 (46.0%)
	Rural	94 (32.5%)
Education level: number (%)	High school degree or less	47 (16.3%)
	Some college without a degree	89 (30.8%)
	Bachelor’s degree or above	153 (52.9%)

**Table 2:** Supplement use pattern secondary to RCC diagnosis among the survey respondents.

Supplement	Female: Number (%)	Male: Number (%)	*p*-Value
Probiotics	31 (21.7%)	39 (26.9%)	0.3
Cannabidiol (CBD) oil or Marijuana	24 (16.8%)	37 (25.5%)	0.07
Vitamin C	20 (14.0%)	34 (23.4%)	0.04
Turmeric or Curcumin	18 (12.6%)	22 (15.1%)	0.5
Vitamin E	11 (7.7%)	11 (7.6%)	0.9

## Data Availability

The original contributions presented in the study are included in the article, further inquiries can be directed to the corresponding author.

## Appendix A

**Survey Questionnaire**

Q1 How do you describe your gender identity?

○ Female
○ Male
○ I prefer not to answer
○ Non-binary
○ Transgender

Q2 Which option best describes your ethnic group or background?

○ White
○ Black or African-American
○ Asian
○ American Indian or Native American
○ Hispanic or Latino
○ Middle Eastern or North African
○ Pacific Islander
○ Prefer not to disclose
○ Other

Q3 Which best describe where you live?

○ Urban
○ Suburban
○ Rural

Q4 What country do you live in?

○ Drop down menu with full list of countries

Q5 What is the highest level of school you have completed or the highest degree you have received?

○ Less than high school degree
○ High school degree or equivalent (e.g., GED)
○ Some college but no degree
○ Associate degree
○ Bachelor degree
○ Graduate degree

Q6 Which of the following treatments have you had? Please specify the order that you received them.

	**1st Treatment**	**2nd Treatment**	**3rd Treatment**	**4th Treatment**	**5th Treatment**	**6th Treatment**	**7th Treatment**	**8th Treatment**	**9th Treatment**	**10th or Later Treatment**	
Opdivo + Yervoy (nivolumab + ipilimumab)											
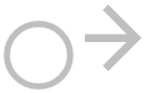
Keytruda + Inlyta (pembrolizumab + axitinib)											
Opdivo + Cabometyx (nivolumab + cabozantinib)											
Keytruda + Lenvima (pembrolizumab + lenvatinib)											
Bavencio + Inlyta (avelumab + axitinib)											
Cabometyx (cabozantinib) single agent											
Lenvima + Afinitor (lenvatinib + everolimus)											
Fotivda (tivozanib)											
Inlyta (axitinib) single agent											
Sutent (sunitinib) single agent											
Votrient (pazopanib) single agent											
Afinitor (everoliumus) single agent											
Other (specify in box below)											

Q7 How long following the discovery of metastatic cancer did you start systemic therapy (drug treatment)?

○ Within 2 weeks
○ Within 4 weeks
○ Within 3 months
○ Within 6 months
○ More than 6 months

Q8 How much money do you spend per month on health related products that are not covered by insurance, such as supplements, vitamins, over the counter medicines.

○ Nothing
○ <$20
○ $20–$50
○ $50–$100
○ $100–$250
○ $250–$500
○ >$500

Q9 Have you taken any of the following alternative supplements as a result of your cancer diagnosis?

○ Marijuana
○ Rick Simpson Oil (RSO)
○ CBD oil
○ Vitamin C
○ Turmeric/Curcumin
○ Fenbendazole
○ Ivermectin
○ Probiotics
○ Vitamin E
○ Other (please specify)

Q10 What statements most accurately reflect your views/experience related to alternative treatments. (Check all that apply).

○ I have tried/would try alternative treatments to help shrink my cancer instead of using approved treatments
○ I have tried/would try alternative treatments in addition to my prescribed cancer drugs to try enhance their effect
○ I have tried/would try alternative treatments to help alleviate side effects or increase quality of life
○ I don’t see any harm in trying alternative treatments
○ I am skeptical of alternative treatments
○ I worry that alternative treatments might cause my cancer to grow
○ I worry that alternative treatments might have a negative impact on my current therapy
○ I worry that alternative treatments might have unknown/negative side effects that would reduce my quality of life

Q11 Do you tell your doctor about supplements you are taking?

○ Always
○ Usually
○ Sometimes
○ Rarely
○ Never

Q12 Have you adopted a different diet as a result of your cancer diagnosis?

○ Ketogenic (low sugar) diet
○ Vegan diet
○ Vegetarian diet
○ Mediterranean diet
○ Other (please specify)
